# Effects of *Lycium barbarum* polysaccharide on the activation of pathogenic CD4^+^ T cells in a mouse model of multiple sclerosis

**DOI:** 10.4103/NRR.NRR-D-24-01093

**Published:** 2025-03-25

**Authors:** Mengdi Guo, Guozhen Deng, Bin Huang, Zhiyong Lin, Xue Yang, Linglin Dong, Zilin Wang, Yi Guo, Ming Yi, Weiyan Wang, Mei-Ling Jiang, Cun-Jin Zhang

**Affiliations:** 1 Department of Neurology, Sichuan Provincial People’s Hospital, University of Electronic Science and Technology of China, Chengdu, Sichuan Province, China; 2 Department of Urology, Institute of Urology, West China Hospital, Sichuan University, Chengdu, Sichuan Province, China; 3 Department of Science and Technology, Sichuan Provincial People’s Hospital, University of Electronic Science and Technology of China, Chengdu, Sichuan Province, China

**Keywords:** AP-1 signaling pathway, experimental autoimmune encephalomyelitis, *Lycium barbarum* glycopeptide, multiple sclerosis, neuroinflammation, nucelar factor-κB signaling pathway, NLRP3 inflammasome, pathogenic CD4^+^ T cells, T helper 1/T helper 17 cell differentiation, Treg polarization

## Abstract

Multiple sclerosis is a severe autoimmune disorder that is mainly mediated by pathogenic cluster of CD4^+^ T cell subsets. Despite advancements in the management of multiple sclerosis, there is a critical need for more effective and safer treatments. In the present study, we administered *Lycium barbarum* glycopeptide to a mouse model of experimental autoimmune encephalomyelitis—an animal model of multiple sclerosis—and evaluated its effects on pathogenic CD4^+^ T cell activation both *in vivo* and *in vitro*. *Lycium barbarum* glycopeptide significantly mitigated the clinical severity of experimental autoimmune encephalomyelitis, as demonstrated by reduced demyelination and neuroinflammation. Moreover, *Lycium barbarum* glycopeptide treatment decreased the infiltration of peripheral leukocytes into the central nervous system and suppressed pro-inflammatory cytokine expression. *Lycium barbarum* glycopeptide also modulated pathogenic CD4^+^ T cell activation by inhibiting T helper 1/T helper 17 cell differentiation while promoting regulatory T cell expansion. Notably, no side effects were observed, suggesting the long-term safety and tolerability of *Lycium barbarum* glycopeptide. Furthermore, RNA sequencing data indicated that *Lycium barbarum* glycopeptide inhibits activator protein-1, an essential regulator of T cell activation and differentiation. This finding was supported by the reversal of T helper/T helper 17 cell response suppression upon AP-1 blockade. Collectively, these results highlight the potential of *Lycium barbarum* glycopeptide as an innovative therapeutic agent for CD4^+^ T cell-associated autoimmune or inflammatory diseases, such as multiple sclerosis.

## Graphical abstract

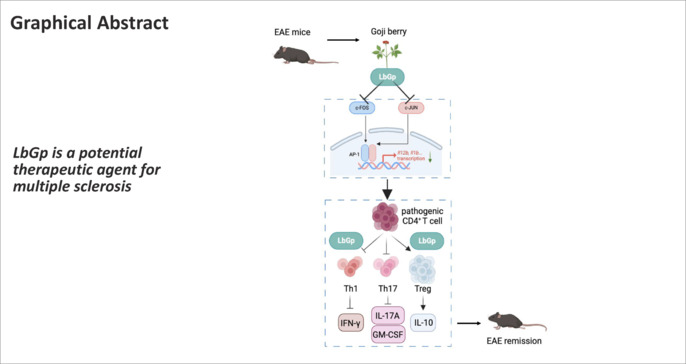

## Introduction

Multiple sclerosis (MS) is a chronic, immune-mediated disorder of the central nervous system (CNS) that primarily affects young adults (Koch-Henriksen and Magyari, 2021; McGinley et al., 2021; Jakimovski et al., 2024). It is characterized by inflammatory demyelination and neuroaxonal degeneration, resulting in a broad spectrum of neurological impairments and accumulating disability (Benedict et al., 2020; Kuhlmann et al., 2023; Jakimovski et al., 2024). The clinical manifestations of MS are highly variable, ranging from mild sensory disturbances to severe motor dysfunction and cognitive impairment. This variability reflects the complex underlying immunopathology in which the activation and infiltration of myelin antigen-autoreactive T cells drive a destructive immune response against CNS myelin, although the precise cause is unknown (Bar-Or and Li, 2021; Liu et al., 2022; Yi et al., 2022). In the pathogenesis of MS, T helper (Th)1 and Th17 cells are particularly prominent in the cerebrospinal fluid of MS patients, where they play crucial roles in initiating inflammatory cascades that compromise blood–brain barrier integrity, activate glia, and result in myelin destruction and neuronal damage. The inflammatory process is not only confined to the CNS but also involves peripheral immune activation, further complicating disease progression and management (Fletcher et al., 2010; Loos et al., 2020; Moser et al., 2020). Although current therapeutic strategies targeting pathogenic T cells have shown efficacy in mitigating disease progression and reducing relapse rates, such treatments are often accompanied by important limitations including systemic immunosuppression, an increased risk of opportunistic infections, and poor patient compliance because of adverse side effects (Vargas and Tyor, 2017; Cortese et al., 2021; McGinley and Cohen, 2021). In summary, despite advances in MS management, the development of safer and more effective therapies targeting pathogenic CD4^+^ T cell responses remains an unmet need.

*Lycium* (*L.*) *barbarum* glycopeptide (LbGp) is a natural active substance that is derived from the fruit of *L. barbarum* (commonly referred to as Goji berries). It has been traditionally used in Chinese medicine for its wide range of health-promoting attributes (Kong et al., 2024; Lakshmanan et al., 2024). In recent years, LbGp has garnered considerable interest for its multifaceted pharmacological properties, which encompass antioxidant, anti-tumor, and neuroprotective effects (Huang et al., 2022; Dai et al., 2023; Xu et al., 2024a). LbGp also reportedly modulates the immune system and reduces inflammation in both systemic and central immune diseases (Cao et al., 2022a; Li et al., 2023b; Xu et al., 2024a). Extensive preclinical and clinical studies have documented the anti-inflammatory properties of LbGp, indicating its potential for modulating immune responses in conditions marked by excess inflammation (Li et al., 2023a, 2024). These properties make LbGp a promising candidate for addressing various pathological conditions, particularly those characterized by immune dysregulation and chronic inflammation. However, despite these promising findings, the therapeutic effects of LbGp in the context of MS/experimental autoimmune encephalomyelitis (EAE, a widely used animal model for MS) have yet to be fully explored. Additionally, previous studies have observed decreased levels of interleukin (IL)-17A—a key cytokine primarily produced by Th17 cells and involved in MS/EAE pathogenesis—in the plasma of patients undergoing LbGp treatment (Li et al., 2024). Nonetheless, the exact mechanisms by which LbGp modulates IL-17 production remain unclear. Understanding these mechanisms may open new avenues for the development of LbGp as a novel therapeutic approach for MS and other pathogenic T cell-mediated conditions.

In the current study, we aimed to assess the therapeutic potential of LbGp in the context of EAE. Our investigation specifically focused on elucidating the mechanisms by which LbGp influences pathogenic CD4^+^ T cell responses, with a particular emphasis on Th1/Th17 subsets, which are implicated in the inflammatory and autoimmune pathogenesis of MS. We also investigated whether LbGp treatment can promote regulatory T (Treg) cell polarization and explored the mechanisms through which LbGp may influence key signaling pathways—particularly the activator protein-1 (AP-1) pathway—in T cell activation and differentiation. The present study sought to provide foundational insights into the potential application of LbGp as a safe and effective therapeutic alternative for MS that directly targets pathogenic immune responses, without the broad immunosuppressive limitations of current therapies.

## Methods

### Animals

Female C57BL/6J mice (specific pathogen-free grade, 23 ± 1 g, 10–12 weeks old) were sourced from GemPharmatech Co., Ltd. (Nanjing, China; license No. SCXK (Chuan) 2020-034) for EAE model induction as previously described (Peruzzotti-Jametti et al., 2024). Upon arrival, animals were acclimatized for at least 1 week prior to the commencement of experimental procedures. The mice were maintained in individually ventilated cages in a sterile environment, with parameters regulated to 22 ± 2°C, 40%–60% relative humidity, and a 12-hour light/dark cycle. Sterile chow diets and autoclaved water were supplied *ad libitum*. To minimize stress and maintain consistent experimental conditions, all handling and procedural manipulations were conducted by a uniform group of trained personnel. All animal experiments were conducted with the approval of the Ethics Committee of Sichuan Provincial People’s Hospital on April 13, 2024 (approval No. 2024574). All experiments were designed and reported according to the Animal Research: Reporting of *In Vivo* Experiments (ARRIVE) guidelines (Percie du Sert et al., 2020).

Mice were randomly divided into four groups: EAE mice treated with normal saline (*n* = 8/9), EAE mice treated with 1 mg/kg LbGp (*n* = 8), EAE mice treated with 5 mg/kg LbGp (*n* = 8/9), and EAE mice treated with 10 mg/kg LbGp (*n* = 8).

### Induction of experimental autoimmune encephalomyelitis models and clinical symptom evaluations

Myelin oligodendrocyte glycoprotein (MOG)35-55 emulsion was prepared and EAE models were induced as previously described (Cao et al., 2022b). The prepared MOG_35–55_ (Baigebio, Hangzhou, China) emulsion was subcutaneously injected at four distinct sites along the dorsal surface of each mouse. Pertussis toxin (500 ng per mouse, List Labs, Campbell, CA, USA) was intraperitoneally administered immediately after immunization and again 48 hours later. Clinical manifestations of EAE were evaluated once daily by trained personnel who were blinded to the experimental conditions. Mice were monitored for the onset and progression of neurological symptoms, which were systematically scored using a standardized 0–5 scale (Fleming et al., 2005). Clinical scoring was performed at consistent times each day to reduce variability and allow for the accurate monitoring of disease progression. Mice exhibiting severe symptoms (scores ≥ 4) were monitored more frequently to ensure timely humane endpoints if necessary.

### *Lycium barbarum* polysaccharide administration

The LbGp powder was a gift from Professor Kwok-Fai So (Li et al., 2022); it was dissolved in normal saline to achieve a homogeneous solution. For *in vivo* studies, mice were administered dissolved LbGp at a weight-based dosage of 1, 5, or 10 mg/kg via oral gavage. The administration was performed once daily and was continued consistently throughout the experimental period. Furthermore, LbGp was dissolved in cell culture at a concentration of 200 μg/mL for *in vitro* experiments to assess its effects under controlled conditions.

### Immunofluorescent staining

Mice were anesthetized with 2% inhaled isoflurane (RWD, Shenzhen, China) and euthanized by carbon dioxide inhalation. Spinal cord tissues fixed in 4% paraformaldehyde were embedded in paraffin and sectioned at a thickness of 5 μm using a microtome. The paraffin sections were then deparaffinized in xylene and rehydrated through a graded series of ethanol solutions. Sections were blocked with 5% bovine serum albumin (Solarbio, Beijing, China) for 1 hour at room temperature. The slides were then incubated with primary antibodies at 4°C overnight. The next day, sections were stained with secondary antibodies for 1.5 hours in darkness at room temperature. All antibodies are listed in **Table 1**. Nuclei were then counterstained with 4′,6-diamidino-2-phenylindole (1:1000, Bioworlde, Nanjing, China) for 15 minutes. Fluorescent images were obtained using a confocal microscope (Zeiss, Oberkochen, Germany), and microglia (ionized calcium-binding adapter molecule 1 [Iba-1]^+^) and astrocyte (glial fibrillary acidic protein [GFAP]^+^) densities were quantified by counting the number of stained positive cells in randomly selected fields of view using ImageJ (version 1.53a, National Institutes of Health, Bethesda, MD, USA) (Schneider et al., 2012).

**Table 1:** Information of antibodies

Antibody	Clone	Dilution	Company	Catalog number	RRID	Application
PerCP/Cyanine5.5 anti-mouse CD45	30-F11	1:200	BioLegend, San Diego, CA, USA	103132	AB_893340	FC
Brilliant Violet 650^TM^ anti-mouse CD3	17A2	1:50	BioLegend	100229	AB_11204249	FC
Alexa Fluor® 488 anti-mouse CD4	GK1.5	1:200	BioLegend	100423	AB_389302	FC
PE anti-mouse/rat/human FOXP3	150D	1:100	BioLegend	320008	AB_492980	FC
Brilliant Violet 421^TM^ anti-mouse CD25	PC61	1:100	BioLegend	102034	AB_10895908	FC
Brilliant Violet 421^TM^ anti-mouse IL-17A	18H10.1	1:100	BioLegend	506926	AB_10900442	FC
PE anti-mouse IFN-γ	XMG1.2	1:100	BioLegend	505808	AB_315401	FC
APC/Cyanine7 anti-mouse CD8a	53-6.7	1:50	BioLegend	100714	AB_312752	FC
PE/Cyanine7 anti-mouse CD45R/B220	RA3-6B2	1:100	BioLegend	103222	AB_313004	FC
Brilliant Violet 421^TM^ anti-mouse Ly-6G	1A8	1:100	BioLegend	127628	AB_10897944	FC
Brilliant Violet 605^TM^ anti-mouse Ly-6C	HK1.4	1:50	BioLegend	128036	AB_2562352	FC
APC anti-mouse/human CD11b	M1/70	1:200	BioLegend	101212	AB_312794	FC
PE anti-mouse F4/80	BM8	1:50	BioLegend	123110	AB_893486	FC
Purified anti-mouse CD16/32	93	1:100	BioLegend	101302	AB_312800	FC
GFAP Mouse mAb	GA5	1:500	Cell Signaling Technology, Danvers, MA, USA,	3670S	AB_561049	IF
Iba1/AIF-1 XP® rabbit mAb	E4O4W	1:500	Cell Signaling Technology	17198S	AB_2820254	IF
Donkey anti-rabbit IgG (H+L) highly cross-adsorbed secondary antibody, Alexa Fluor^TM^ 594	/	1:500	Invitrogen, Carlsbad, CA, USA	A-21207	AB_141637	IF
Donkey anti-mouse IgG (H+L) highly cross-adsorbed secondary antibody, Alexa Fluor^TM^ Plus 488	/	1:500	Invitrogen	A-32766	AB_2762823	IF
NLRP3 rabbit mAb	D4D8T	1:1000	Cell Signaling Technology	15101S	AB_2722591	WB
Anti-NF-kB p65 antibody	E379	1:1000	Abcam, Shanghai, China	ab32536	AB_776751	WB
Phospho-NF-κB p65 (Ser468) recombinant antibody	6N1	1:2000	Proteintech, Wuhan, China	82335-1-RR	AB_3083091	WB
c-FOS mouse monoclonal antibody	1G2C5	1:10000	Proteintech	66590-1-Ig	AB_2881950	WB
Jun rabbit polyclonal antibody	/	1:2000	Proteintech	24909-1-AP	AB_2860574	WB
Phospho-IkB alpha (Ser32/36) recombinant rabbit antibody	1G21	1:5000	Proteintech	82349-1-RR	AB_3073626	WB
IkB alpha rabbit polyclonal antibody	/	1:5000	Proteintech	10268-1-AP	AB_2151423	WB
Beta actin mouse monoclonal antibody	2D4H5	1:20000	Proteintech	66009-1-Ig	AB_2687938	WB
Goat anti-rabbit IgG (H+L) HRP	/	1:5000	Bioworlde, Nanjing, China	BS13278	AB_2773728	WB
Goat anti-mouse IgG (H+L) HRP	/	1:5000	Bioworlde	BS12478	AB_2773727	WB

FC: Flow cytometry; IF: immunofluorescence staining; WB: western blotting.

### Hematoxylin and eosin staining

Spinal cord sections were stained with hematoxylin (Solarbio) for 5 minutes, followed by a brief rinse in tap water. Sections were then differentiated in 1% acid alcohol, blued in lithium carbonate solution (Solarbio), and counterstained with eosin (Solarbio) for 2 minutes. After staining, the sections were dehydrated through an ascending ethanol series, cleared in xylene, and mounted with a coverslip using a synthetic resin. The stained sections were examined under a light microscope (Nikon, Tokyo, Japan) to evaluate immune cell infiltration. Cell densities were quantified using ImageJ.

### Luxol fast blue staining

Spinal cord sections were incubated in Luxol fast blue (LFB) solution (Solarbio) overnight at 56°C to stain the myelin sheaths. Subsequently, excess stain was removed by rinsing in 95% ethanol and then distilled water. Demyelination areas were identified as regions where the LFB staining was significantly reduced or absent using ImageJ. Histological scoring was performed using a 0–3 scale as previously reported (Ma et al., 2021).

### Preparation of single-cell suspensions

EAE mice were euthanized with carbon dioxide inhalation and their organs were quickly collected in ice-cold phosphate-buffered saline. Blood samples were collected via cardiac puncture. Brain tissue was mechanically dissociated into small pieces and homogenized in complete RPMI-1640 medium (ExCell Bio, Soochow, China). To isolate immune cells, the cell suspension was subjected to a 70%/100% Percoll (Cytiva, Logan, UT, USA) gradient centrifugation at 500 × *g* for 25 minutes at room temperature. The immune cell layer—located at the Percoll layer interface—was carefully collected, washed with fluorescence-activated cell sorting buffer, and resuspended for further analysis. The lymph nodes and spleen were excised and mechanically dissociated using a 70-μm cell strainer (Biosharp, Hefei, China). To remove erythrocytes, the cell suspension was treated with ammonium-chloride-potassium lysis buffer (Solarbio) for 5 minutes, followed by neutralization with fluorescence-activated cell sorting buffer.

### Flow cytometry

To ensure specific staining and minimize non-specific binding, immune cells from the murine brain, spleen, lymph node, blood, and thymus were initially blocked with anti-mouse CD16/32 for 5 minutes at 4°C (to inhibit Fc receptor binding). Cells were then incubated with surface marker antibodies for 40 minutes at 4°C in darkness (to maintain the fluorescent integrity). Next, cells were washed and fixed using 1× permeabilization buffer (eBioscience, San Diego, CA, USA) to allow intracellular access for further staining. Intracellular cytokines were stained with the appropriate antibodies for 60 minutes at room temperature in darkness (to avoid photobleaching). When the staining was complete, samples were analyzed using a flow cytometer (Agilent, Hangzhou, China) and data acquisition was performed using NovoExpress software (version 1.4.0, ACEA Biosciences, San Diego, CA, USA). Antibody details, including clone numbers, concentrations, and fluorophores, are provided in **Table 1**. The combination of surface and intracellular staining allowed for a comprehensive analysis of both cell phenotypes and cytokine production in the different experimental groups.

### Annexin V and propidium iodide staining

The staining procedure was performed according to the manufacturer’s instructions (BioLegend, San Diego, CA, USA). CD4^+^ T cells were recognized as live (propidium iodide [PI]^–^ Annexin V [AV]^–^), apoptotic (PI^–^AV^+^), or necrotic/dead (PI^+^AV^+^) cells using flow cytometric analysis.

### Quantitative polymerase chain reaction

Total RNA was isolated from murine spinal cords or CD4^+^ T cells using TRIzol reagent, and was then reverse transcribed to complementary DNA (Vazyme, Nanjing, China). Quantitative polymerase chain reaction was conducted using an AceQ Universal SYBR qPCR Mix (Vazyme) on a LightCycler polymerase chain reaction instrument (Roche, Basel, Switzerland). Primer sequences are shown in **Table 2**.

**Table 2:** The primer sequences for quantitative polymerase chain reaction

Target gene	Sequence (5′ to 3′)
*Actb*	F: TGT CCA CCT TCC AGC AGA TGT
	R: AGC TCA GTA ACA GTC CGC CTA GA
*Tnfa*	F: CCA CCA CGC TCT TCT GTC TA
	R: GAT CTG AGT GTG AGG GTC TGG
*Il1b*	F: CCA TCC TCT GTG ACT CAT GGG
	R: TCA GCT CAT ATG GGT CCG AC
*Il6*	F: GAC AAA GCC AGA GTC CTT CAG AGA G
	R: CTA GGT TTG CCG AGT AGA TCT C
*Ifng*	F: ATG AAC GCT ACA CAC TGC ATC
	R: CCA TCC TTT TGC CAG TTC CTC
*Il17a*	F: CTC CAC CGC AAT GAAGAC
	R: CTT TCC CTC CGC ATT GAC
*Cxcl1*	F: CCA GAG CTT GAA GGT GTT GC
	R: TGA ACC AAG GGA GCT TCA G
*Cxcl9*	F: AAA CCT GCC TAG ATC CGG AC
	R: CGA CTT TGG GGT GTT TTG GG
*Tbx21*	F: CAA CAA CCC CTT TGC CAA AG
	R: TCC CCC AAG CAG TTG ACA GT
*Stat1*	F: GCC TCT CAT TGT CAC CGA AGA AC
	R: TGG CTG ACG TTG GAG ATC ACC A
*Stat4*	F: TCA GTG AGA GCC ATC TTG GAG G
	R: TGT AGT CTC GCA GGA TGT CAG C
*Rora*	F: GAA CAC CTT GCC CAG AAC AT
	R: AGC TGC CAC ATC ACC TCT CT
*Runx1*	F: TAC CTG GGA TCC ATC ACC TC
	R: GAC GGC AGA GTA GGG AAC TG
*Il12b*	F: TTG AAC TGG CGT TGG AAG CAC G
	R: CCA CCT GTG AGT TCT TCA AAG GC
*Fos*	F: GGG AAT GGT GAA GAC CGT GTC A
	R: GCA GCC ATC TTA TTC CGT TCC C
*Jun*	F: CAG TCC AGC AAT GGG CAC ATC A
	R: GGA AGC GTG TTC TGG CTA TGC A
*Ptgs2*	F: GCG ACA TAC TCA AGC AGG AGC A
	R: AGT GGT AAC CGC TCA GGT GTT G
*Egr1*	F: AGC GAA CAA CCC TAT GAG CAC C
	R: ATG GGA GGC AAC CGA GTC GTT T
*Csf2*	F: TCG TCT CTA ACG AGT TCT CCT T
	R: CGT AGA CCC TGC TCG AAT ATC T
*Il2*	F: AGA TGA ACT TGG ACC TCT GCG
	R: AAA GTC CAC CAC AGT TGC TG
*Il21*	F: CGC CTC CTG ATT AGA CTT CG
	R: GCC CCT TTA CAT CTT GTG GA

F: Forward; R: reverse.

### Body weight monitoring

Mice were administered either normal saline or 5 mg/kg LbGp (*n* = 5). The body weights of mice were measured using a scale and recorded weekly for four consecutive weeks.

### Spleen weight and lymph node size measurements

Mice were euthanized by CO_2_ inhalation, and the spleen and inguinal lymph nodes were removed. The spleen weights and lymph node sizes were measured and recorded.

### Magnetic-activated cell sorting (MACS®)

CD4^+^ T cells or CD4^+^CD62L^high^CD44^low^ naïve T cells from the murine spleen and lymph nodes were isolated using a commercial kit (Miltenyi Biotec, Bergisch Gladbach, Germany). Single-cell suspensions were incubated with biotin-conjugated antibodies before being subjected to magnetic separation using MACS technology. This allowed for the selective isolation of CD4^+^ T cells by retaining them in the magnetic field while non-target cells were washed away. For naïve T cell isolation, further selection was performed based on CD62L^high^ and CD44^low^ expression. Following isolation, isolated population purity was confirmed via flow cytometric analysis; this typically exceeded 95%, thus ensuring high-quality samples for downstream applications such as functional assays or gene expression studies.

### *In vitro* Th1/Th17 cell differentiation assay

Naïve CD4^+^ T cells that had been purified using MACS were stimulated with 5 μg/mL anti-CD3 and 2 μg/mL anti-CD28. Th1 cell differentiation was induced by adding IL-12 (20 ng/mL, R&D Systems, Minneapolis, MN, USA) and anti-IL-4 antibody (5 μg/mL, R&D Systems) to the culture. Th17 polarization was induced using IL-6 (20 ng/mL, R&D Systems), IL-1β (10 ng/mL, R&D Systems), IL-23 (20 ng/mL, R&D Systems), anti-interferon-γ (IFN-γ; 5 μg/mL, R&D Systems), and anti-IL-4 antibody (5 μg/mL). Cells were cultured for 3–5 days with vehicle (dimethyl sulfoxide) or the AP-1 inhibitor T-5224 (10 μM dissolved in dimethyl sulfoxide; MCE, Shanghai, China) before being stimulated with phorbol 12-myristate 13-acetate (PMA; 1 mg/mL, MCE), ionomycin (500 μg/mL, MCE), and brefeldin A (5 μg/mL, MCE) for 3–4 hours to enhance cytokine production. Intracellular staining was conducted after fixing the cells with paraformaldehyde and permeabilizing them using a permeabilization buffer. The cells were labeled with fluorochrome-conjugated antibodies specific to IFN-γ (BioLegend) and IL-17A (BioLegend). Flow cytometry was used to analyze the stained cells, allowing for the quantification of the percentage of cells producing these cytokines.

### MOG_35–55_ peptide restimulation assay

Splenocytes or lymph node cells were harvested at the pre-onset stage of EAE (8 days post-immunization). For antigen-specific restimulation, the isolated cells were plated in 12-well flat-bottom plates at a density of 5 × 10^6^ cells per well in 2 mL of complete RPMI-1640 medium (ExCell Bio). Cells were then restimulated for 72 hours with 25 μg/mL MOG_35–55_ peptide (MEVGWYRSPFSRVVHLYRNGK, BaigeBio, Hangzhou, China). To induce Th1/Th17 cell polarization, 20 ng/mL of IL-12 or 10 ng/mL of IL-1β and 20 ng/mL of IL-23 (R&D Systems) were added to the cell culture medium (ExCell Bio), respectively. Intracellular cytokine and forkhead box P3 (FOXP3) staining was performed as described in ‘*In vitro* Th1/Th17 cell differentiation assay’ before being detected using flow cytometric analysis (Cao et al., 2022b).

### Enzyme-linked immunosorbent assay

Cytokine concentrations of MOG-restimulated cell supernatants were measured using commercially available enzyme-linked immunosorbent assay kits (IL-17A and IFN-γ, Invitrogen, Carlsbad, CA, USA; granulocyte-macrophage colony-stimulating factor and IL-10, BioLegend). Optical density values were measured at 450 nm and sample concentrations were determined via comparisons with the standard curve.

### RNA sequencing analysis

Total RNA was isolated from MACS-purified CD4^+^ T cells using a commercial RNA extraction kit (Vazyme, Nanjing, China). RNA sequencing (RNA-seq) libraries were prepared, and sequencing was performed using an Illumina platform (Liebing Technology Biomedical Co., Ltd., Shangrao, China). DESeq2 (https://bioconductor.org/packages/release/bioc/html/DESeq2.html) was used to analyze differential gene expression; significant differentially expressed genes (DEGs) were identified by a false discovery rate (FDR) < 0.05 and an absolute fold change > 1.5. The top 20 enriched gene sets were identified using gene set enrichment analysis with the following criteria: absolute value of normalized enrichment score (|NES|) > 1.5, *P* < 0.05, and FDR < 0.10. Heatmaps were generated to illustrate significant DEGs associated with specific signaling pathways.

### Western blotting

Protein samples from murine lymph node were separated using 7.5%–12.5% sodium dodecyl sulfate-polyacrylamide gel electrophoresis (Yamei, Shanghai, China), transferred to polyvinylidene difluoride membranes (MerckMillipore, Bedford, MA, USA), and blocked with 5% milk powder in 1× Tris-buffered saline with Tween-20 (Biosharp, Hefei, China). The membranes were then sequentially incubated with specific primary antibodies at 4°C overnight and secondary antibodies at room temperature for 2 hours. All antibodies are shown in **Table 1**. Gels were visualized using a Gel-Pro system (Tanon, Shanghai, China) and gray values were quantified using ImageJ. The grayscale intensity of the target protein was normalized against β-actin, and the level of protein phosphorylation was normalized to the corresponding total protein.

### Statistical analysis

Although statistical methods were not used to determine the sample size, it was comparable with that reported in a previous study (Cao et al., 2022b). Data acquisition and evaluation were blinded. All analyses were conducted using GraphPad Prism 9.0.0 (GraphPad Software, Boston, MA, USA, www.graphpad.com) and values are presented as the mean ± standard error of the mean. The *P*-values for clinical scores were determined via two-way analysis of variance. One-way analysis of variance was used for comparisons between multiple groups. Post hoc comparisons were performed using the Bonferroni test. The unpaired Student’s *t*-test was used for two-group analyses. The significance threshold was set as *P* < 0.05.

## Results

### Daily oral administration of *Lycium barbarum* glycopeptide ameliorates experimental autoimmune encephalomyelitis clinical course and neuroinflammation

**Figure 1:**
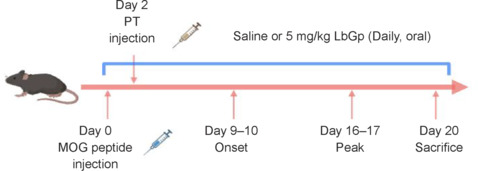
Schematic representation of the experimental EAE paradigm and treatment protocol. An active EAE model was induced in 3-month-old female C57BL/6J mice by injection of MOG35-55 emulsion. Next, mice were administered either LbGp or normal saline. Clinical scores were used to monitor EAE progression and mice were sacrificed 20 days post-immunization to assess disease pathology. Created with BioRender.com. EAE: Experimental autoimmune encephalomyelitis; LbGp: *Lycium barbarum* polysaccharide; MOG: myelin oligodendrocyte glycoprotein; PT: pertussis toxin.

To assess the influence of LbGp on EAE severity and CNS pathology, an active EAE model was induced in 3-month-old female C57BL/6J mice using MOG_35–55_ emulsion injection. A dose-response test was then conducted to determine the optimal therapeutic dosage (**Additional Figure 1**). Although the 1 mg/kg dose had no alleviating effects in EAE mice, the 5 mg/kg treatment dose significantly improved disease progression (**Additional Figure 1**). However, there was no difference between the 10 and 5 mg/kg doses, suggesting that an increased dosage does not further improve disease progression. In subsequent experiments, mice were administered either 5 mg/kg of LbGp or an equivalent volume of normal saline (200 μL) (**Figure 1**). Clinical scores were used to monitor EAE progression, and mice were sacrificed 20 days post-immunization to assess disease pathology.

**Figure 2:**
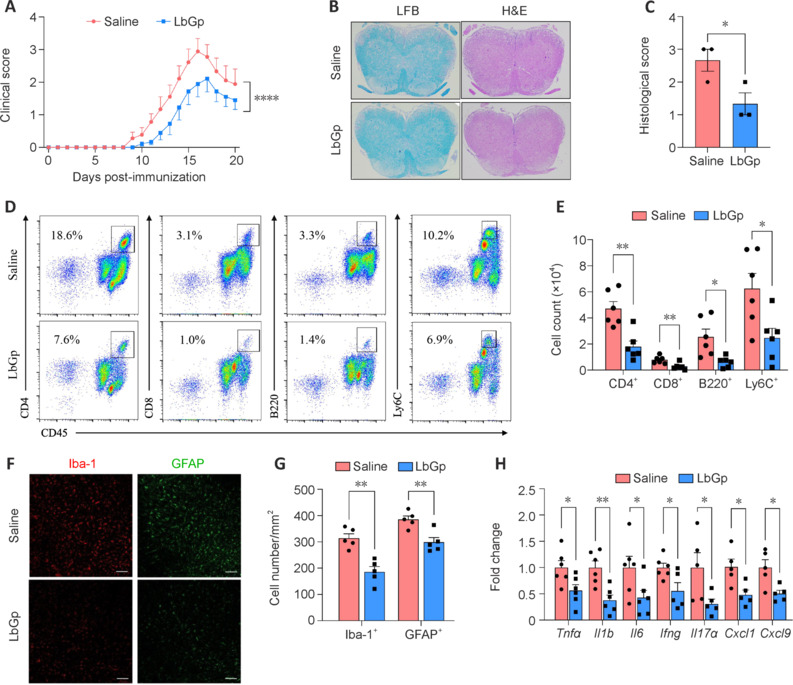
Daily oral administration of LbGp significantly ameliorates EAE pathology and mitigates neuroinflammation. (A) Clinical scores of EAE mice administered with LbGp (5 mg/kg, oral gavage) or normal saline control once daily (*n* = 9 per group). (B) Representative H&E- and LFB-stained sections of spinal cord lesions at the peak stage of EAE mice treated with normal saline or 5 mg/kg LbGp. EAE mice treated with 5 mg/kg LbGp showed a significant decrease in demyelination and infiltrating inflammatory cells compared with the normal saline group. (C) Histological score of spinal cord sections from EAE mice receiving the indicated treatments (*n* = 3 per group). (D, E) Flow cytometric analysis of infiltrating immune cells from the brains of EAE mice receiving the indicated treatments (*n* = 6 per group). Data are presented as representative plots (D) and absolute cell numbers (E). (F) Immunofluorescent staining of Iba-1^+^ (Alexa Fluor 594, red) and GFAP^+^ (Alexa Fluor 488, green) cells in spinal cord sections from EAE mice treated with normal saline or 5 mg/kg LbGp. EAE mice treated with LbGp showed lower densities of Iba-1^+^ and GFAP^+^ cells in the spinal cord sections compared with the normal saline group. Scale bars: 100 μm. (G) Statistical comparison of Iba-1^+^ and GFAP^+^ cell densities (*n* = 5 per group). (H) mRNA expression levels of inflammatory markers in the CNS, assessed at the peak of disease (*n* = 6 per group, normalized to *Actb* mRNA levels). Error bars indicate the mean ± standard error of the mean. *P < 0.05, **P < 0.01, ****P < 0.0001. Actb: β-Actin; CNS: central nervous system; EAE: experimental autoimmune encephalomyelitis; GFAP: glial fibrillary acidic protein; H&E: hematoxylin and eosin; Iba-1: ionized calcium-binding adapter molecule 1; LbGp: *Lycium barbarum* polysaccharide; LFB: Luxol fast blue.

Daily oral LbGp administration significantly reduced the clinical severity of murine EAE, as indicated by lower clinical scores compared with the control group (**Figure 2A**). Hematoxylin and eosin and Luxol fast blue staining revealed a significant decrease in demyelination and infiltrating inflammatory cells in LbGp-treated mice, indicating reduced neuropathological phenotypes (**Figure 2B** and **C**). Flow cytometric analysis confirmed a significant reduction in the infiltration of CD4^+^ T cells, CD8^+^ T cells, B cells (CD45^+^), and monocytes (CD45^+^ lymphocyte antigen 6C [LY6C]^+^) in the brains of LbGp-treated mice (**Figure 2D** and **E**). This reduction in immune cell infiltration indicates that LbGp may effectively suppress the EAE-associated inflammatory response. Consistent with this idea, immunofluorescent staining revealed decreased levels of microglia and astrocytes, as indicated by lower densities of Iba-1^+^ and GFAP^+^ cells in the spinal cord sections (**Figure 2F** and **G**). Furthermore, LbGp treatment led to downregulated levels of inflammatory gene mRNA within the CNS (**Figure 2H**), including of cytokines that promote Th17 cell expansion (*Il1b* and *Il6*), cytokines released by Th1 and Th17 cells (*Tnfα*, *Ifng*, and *Il17a*), and chemokines (*Cxcl1* and *Cxcl9*). Overall, these findings suggest that LbGp plays a protective role in EAE progression and neuroinflammation, highlighting its potential as a therapeutic agent for managing autoimmune neuroinflammatory conditions.

### *Lycium barbarum* glycopeptide is well tolerated over long-term treatment in mice

Previous research has indicated the potential therapeutic benefits and safety of LbGp in a variety of animal species and mouse models, highlighting its promise for modulating immune responses without severe side effects (Wang et al., 2021; Song et al., 2022; Zheng et al., 2023). However, the long-term effects, particularly in terms of immunotoxicity and adverse reactions following prolonged LbGp administration in C57BL/6J mice, have not yet been thoroughly investigated. To address this gap, we evaluated the safety profile of LbGp with respect to its potential toxic effects and impacts on the immune system after extended use. Female C57BL/6J mice were administered a daily oral dose of 5 mg/kg LbGp (or an equivalent volume of normal saline) via gavage for 4 weeks. Throughout the treatment period, body weight was monitored as a basic indicator of overall health. The data revealed no significant weight loss in any of the groups, suggesting that LbGp is well tolerated at the given dosage (**Additional Figure 2A**). To further assess the potential immunotoxicity of LbGp, flow cytometry was performed to analyze the frequency and counts of various immune cell subtypes in the blood, inguinal lymph nodes, spleen, and thymus. The number of CD45^+^ leukocytes was not significantly affected by LbGp treatment (**Additional Figure 2B**). Similarly, the subpopulations of myeloid and lymphoid lineages, such as dendritic cells (CD11c^+^), neutrophils (LY6G^+^), monocytes (LY6C^+^), macrophages (F4/80^+^), T cells (CD3^+^), B cells (CD19^+^), CD4^+^ T cells, and CD8^+^ T cells, showed no significant changes (**Additional Figure 2C–F**). These findings suggest that LbGp does not disrupt the homeostasis of peripheral immune cells or induce immunosuppression or hyperactivation within the studied time frame. Additionally, hematoxylin and eosin staining demonstrated no histopathological changes in the liver, kidney, spleen, or inguinal lymph nodes, further supporting the conclusion that LbGp is non-toxic and well tolerated under the tested conditions (**Additional Figure 2G**). Overall, these data imply that LbGp is well tolerated as a long-term treatment in C57BL/6J mice.

### *Lycium barbarum* glycopeptide modulates the frequency of Th1/Th17 and Tregs in experimental autoimmune encephalomyelitis models

**Figure 3:**
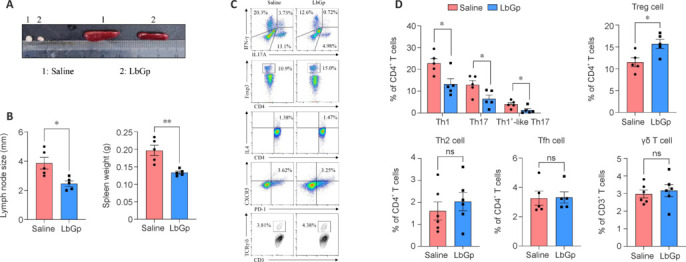
LbGp modulates the frequency of CD4^+^ T cell subsets in EAE models. Three-month-old female C57BL/6J mice were euthanized 8 days post-immunization with MOG_35–55_. (A, B) Lymph node sizes and spleen weights were measured in EAE mice across the different groups (*n* = 5 per group). (C, D) Flow cytometric analysis was conducted to determine the proportions of IFN-γ^+^ (Th1), IL-17A^+^ (Th17), FOXP3^+^ (Treg), IL-4^+^ (Th2), CXCR5^+^PD-1^+^ (Tfh), CD3^+^TCRγ/δ^+^ (γδ T cells), and IFN-γ^+^IL-17A^+^ T cells in the murine spleen (*n* = 5 per group). Error bars indicate the mean ± standard error of the mean. *P < 0.05, **P < 0.01. CD: Cluster of differentiation; CXCR5: C–X–C chemokine receptor type 5; EAE: experimental autoimmune encephalomyelitis; FOXP3: forkhead box P3; IFN-γ: interferon-γ; IL: interleukin; LbGp: Lycium barbarum polysaccharide; MOG: myelin oligodendrocyte glycoprotein; ns: not significant; PD-1: programmed cell death protein 1; TCR: T cell receptor; Tfh: T follicular helper cell; Th: T helper cell; Treg: regulatory T cell.

As previously elucidated, the activation of myelin-specific T cells is a hallmark of MS (Angiari et al., 2020; Cruciani et al., 2021). The pre-onset stages of EAE are predominantly characterized by the clonal expansion of CD4^+^ T cells and their polarization into Th1/Th17 effector cells within peripheral lymphoid organs, which then facilitates immune priming and subsequent central infiltration by pathogenic leukocytes (Damasceno et al., 2020; Gao et al., 2023). Considering that LbGp administration delays the onset of the EAE clinical course (**Figure 2A**), we hypothesized that LbGp might inhibit the activation and pathogenic polarization of peripheral CD4^+^ T cells. To test this hypothesis, we euthanized mice at the pre-onset stage of EAE (8 days post-immunization), measured spleen weight and lymph node size, and assessed the frequency of Th1, Th17, and Tregs in the murine spleen. LbGp treatment effectively reduced lymph node size and spleen weight (**Figure 3A** and **B**), indicating a decreased clonal expansion of MOG-reactive CD4^+^ T cells. LbGp administration also inhibited the conversion of cells into pathogenic Th1, Th17, and IFN-γ^+^IL-17A^+^ effector T cells and significantly enhanced the properties of immunosuppressive Tregs (**Figure 3C** and **D**). By contrast, there were no significant differences observed in the proportions of Th2, T follicular helper, and γδ T cells in the draining lymph nodes (**Figure 3C** and **D**). These findings suggest that LbGp can rebalance the dysregulated CD4^+^ T cell subsets in EAE.

### *Lycium barbarum* glycopeptide constrains Th1/Th17 responses and enhances Treg polarization *in vitro*

**Figure 4:**
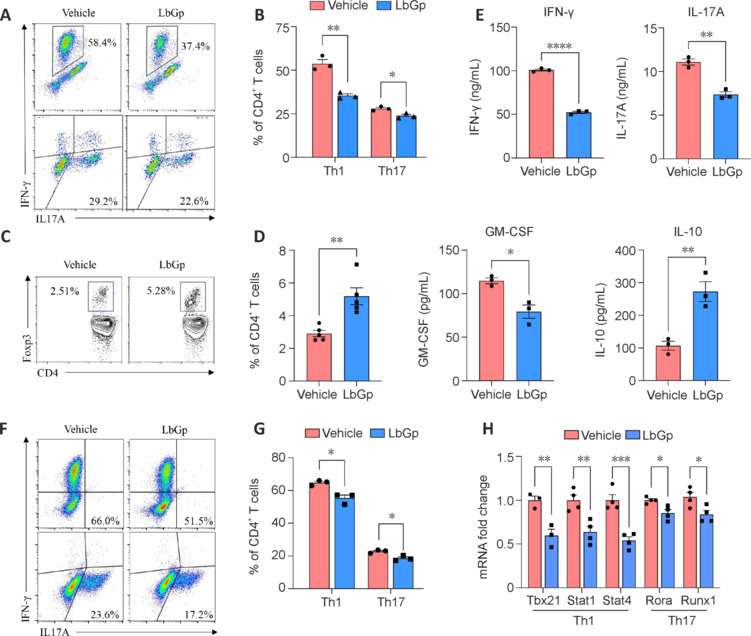
LbGp constrains Th1/Th17 responses and enhances Treg polarization *in vitro*. (A–D) Quantification of the percentages of Th1, Th17, and Tregs in MOG-restimulated lymph node cells treated with either vehicle or LbGp for 2–4 days (*n* = 3 per group). (E) The concentrations of IFN-γ, IL-17A, GM-CSF, and IL-10 in MOG-restimulated cell supernatants were measured using ELISA (*n* = 3 per group). (F, G) The impact of LbGp on Th1/Th17 differentiation in naïve CD4^+^ T cells was assessed *in vitro* (*n* = 3 per group). (H) The quantification of mRNA levels of Th1/Th17 signature transcription factors was performed using quantitative polymerase chain reaction (*n* = 3 per group). Error bars indicate the mean ± standard error of the mean. *P < 0.05, **P < 0.01, ***P < 0.001, ****P < 0.0001. CD: Cluster of differentiation; ELISA: enzyme-linked immunosorbent assay; GM-CSF: granulocyte-macrophage colony-stimulating factor; IFN-γ: interferon-γ; IL: interleukin; LbGp: *Lycium barbarum* polysaccharide; MOG: myelin oligodendrocyte glycoprotein; Th: T helper cell; Treg: regulatory T cell.

To elucidate the role of LbGp in CD4^+^ T cell subset polarization, lymphocytes from EAE mice were restimulated with MOG_35–55_ peptide and exposed to Th1/Th17-conditioned medium *in vitro*. A concentration of 200 μg/mL LbGp, which has previously been used as an effective, non-toxic dose for myeloid cells, was applied for *in vitro* treatment (Jiang et al., 2023). AV and PI staining confirmed no significant impact on cell viability at this concentration (**Additional Figure 3**). Similarly, LbGp treatment led to a profound reduction of Th1/Th17 polarization and enhancement of Treg polarization (**Figure 4A–D**). This shift in cell polarization was accompanied by a marked decrease in the production of pro-inflammatory cytokines IFN-γ, IL-17A, and granulocyte-macrophage colony-stimulating factor, all of which are hallmarks of Th1 and Th17 cell activity. By contrast, we observed an increase in the production of IL-10, a cytokine that is primarily associated with anti-inflammatory Treg function (Chaudhry et al., 2011; **Figure 4E**). These results suggest that LbGp not only suppresses the differentiation of pathogenic CD4^+^ T cell subsets but also promotes an immunoregulatory environment that may help to alleviate autoimmune inflammation.

Next, we investigated whether LbGp directly affects the differentiation of Th1 and Th17 cells. Upon polarization of murine naïve T cells toward Th1/Th17 phenotypes, LbGp markedly inhibited the generation of Th1/Th17 cells, as revealed by reduced percentages of IFN-γ^+^/IL-17A^+^ CD4^+^ T cells following LbGp treatment (**Figure 4F** and **G**). These results further corroborate the notion that LbGp exerts a potent suppressive effect on the development of pathogenic T cell subsets. To gain deeper mechanistic insights into how LbGp influences the differentiation of these pathogenic T cell subsets, we assessed the expression levels of key transcription factors involved in Th1 and Th17 cell polarization. The mRNA levels of essential transcription factors for Th1 differentiation, including Tbx21, Stat1, and Stat4, were significantly downregulated in LbGp-treated cells. Similarly, critical transcription factors for Th17 cell differentiation, such as Rora and Runx1, also showed notably decreased expression following LbGp treatment (Oestreich and Weinmann, 2012; Hang et al., 2019; **Figure 4H**). Collectively, these findings confirm that LbGp treatment limits the pathogenic potential of CD4^+^ T cells *in vitro*, favoring a shift away from Th1 and Th17 cells and toward Treg cells.

### *Lycium barbarum* glycopeptide attenuates inflammatory gene expression profiles and key signaling pathways required for Th1/Th17 responses during experimental autoimmune encephalomyelitis

**Figure 5:**
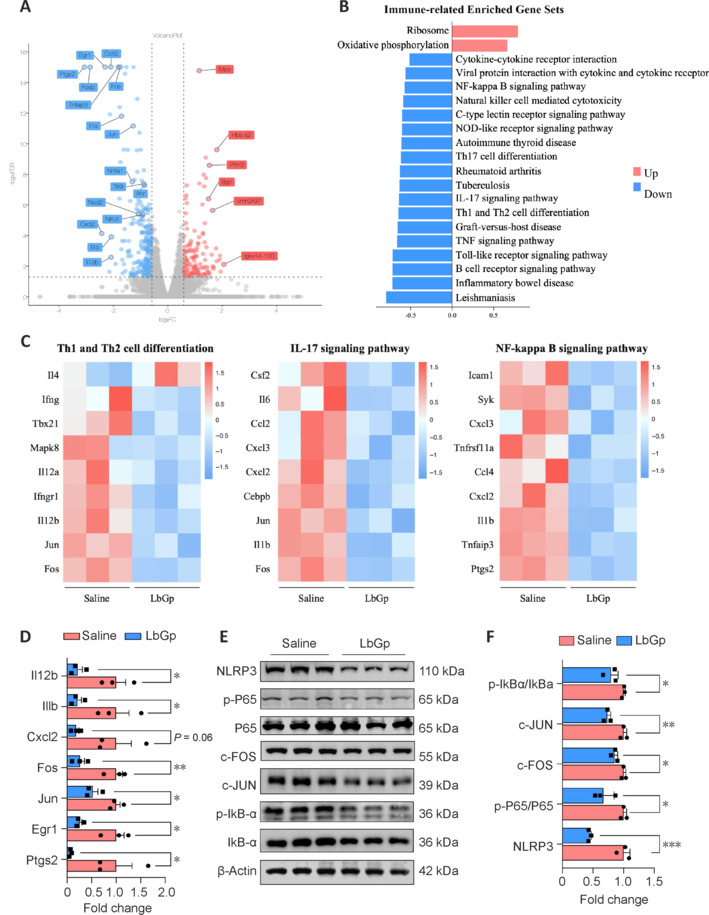
LbGp attenuates inflammatory gene expression profiles and key signaling pathways required for Th1/Th17 responses during EAE. (A) DEGs are summarized in volcano plots (FC > 1.5, *P* < 0.05). (B) The top 20 immune-related enriched gene sets were identified based on GSEA (|NES|> 1.5, *P* < 0.05, FDR < 0.10). (C) Heatmaps illustrating significant DEGs associated with Th1 and Th2 cell differentiation, the IL-17 signaling pathway, and the NF-κB signaling pathway were generated. (D) Validation of the transcriptome sequencing results by quantitative polymerase chain reaction (*n* = 3 per group). (E, F) The activation of NF-κB signaling pathways and the expression of NLRP3, c-FOS, and c-JUN were assessed via western blotting. Error bars indicate the mean ± standard error of the mean. *P < 0.05, **P < 0.01, ***P < 0.001. DEGs: Differentially expressed genes; EAE: experimental autoimmune encephalomyelitis; FC: fold change; FDR: false discovery rate; GSEA: gene set enrichment analysis; LbGp: *Lycium barbarum* polysaccharide; NES: normalized enrichment score; NF-κB: nuclear factor kappa-light-chain-enhancer of activated B cells; NLRP3: NOD-like receptor family pyrin domain containing 3.

The aforementioned results indicate that the suppression of pathogenic CD4^+^ T cell responses by LbGp is crucial for mitigating T cell-mediated autoimmunity. To further explore the molecular mechanisms underlying the immunomodulatory effects of LbGp, we conducted bulk RNA-seq on MACS-purified CD4^+^ T cells from EAE mice treated with either normal saline or LbGp. This approach allowed us to examine LbGp-induced transcriptomic changes and identify DEGs that may provide insights into how LbGp modulates autoimmune responses. The DEGs were selected based on stringent criteria (FDR < 0.05 and fold change ≥ 1.5) (**Figure 5A**). Notably, we observed the significant downregulation of genes related to T cell activation (*Jun*, *Fos*, and *Fosb*), inflammatory T cell responses (*Ptgs2*, *Nod2*, *Nlrc4*, and *Tlr9*), Th1/Th17 cell differentiation (*Ahr*, *Egr1*, *Il1b*, and *Il12b*), and T cell migration (*Cxcl2* and *Ccrl2*). The top 20 enriched gene sets were then identified using gene set enrichment analysis (criteria: |NES| > 1.5, *P* < 0.05, and FDR < 0.10). LbGp negatively regulated gene sets associated with CD4^+^ T cell activation, polarization, infiltration, cytokine and chemokine production, and key adaptive immune response signaling pathways (**Figure 5B**). This finding suggests that LbGp not only affects the activation of CD4^+^ T cells but also disrupts the regulatory networks responsible for maintaining their pro-inflammatory functions.

Heatmaps were generated to illustrate the significant DEGs associated with the T cell differentiation, IL-17 signaling, and nuclear factor (NF)-κB pathways to better understand the specific pathways involved in the therapeutic effects of LbGp (**Figure 5C**). The important roles of these pathways in the development and progression of EAE are well established in the literature. The downregulation of genes within these pathways suggests that LbGp specifically targets signaling mechanisms that are crucial for the pathogenicity of Th1 and Th17 cells. Given the central role of these pathways in driving inflammation and autoimmunity, their suppression by LbGp points to a potential mechanism underlying its broad immunomodulatory effects. To validate the transcriptomic data, we performed quantitative polymerase chain reaction on several key genes that were identified through RNA-seq. LbGp treatment significantly altered the expression levels of these genes, corroborating the findings from our RNA-seq analysis (**Figure 5D**). LbGp treatment also significantly altered the protein expression levels of phosphorylated (p)-NF-κB p65; p-NF-kappa-B inhibitor alpha (IkB-α); NACHT, LRR and PYD domains-containing protein 3 (NLRP3); c-FOS; and c-JUN in lymph node samples from EAE mice (**Figure 5E** and **F**). These proteins are essential for activating signaling pathways that are crucial for Th1/Th17 responses (Mc Guire et al., 2013; Arbore et al., 2016; Martin et al., 2016; Takeuchi et al., 2024). In summary, our findings indicate that LbGp treatment ameliorates gene expression profiles and key signaling pathways that are required for the pathogenicity of CD4^+^ T cells in EAE. By inhibiting crucial pathways such as the NF-κB, IL-17 signaling, and T cell activation pathways, LbGp may effectively suppress the differentiation and function of Th1 and Th17 cells, thereby mitigating the autoimmune response.

### Suppressive effects of LbGp on Th1/Th17 cell differentiation are blocked by AP-1 inhibitor

**Figure 6:**
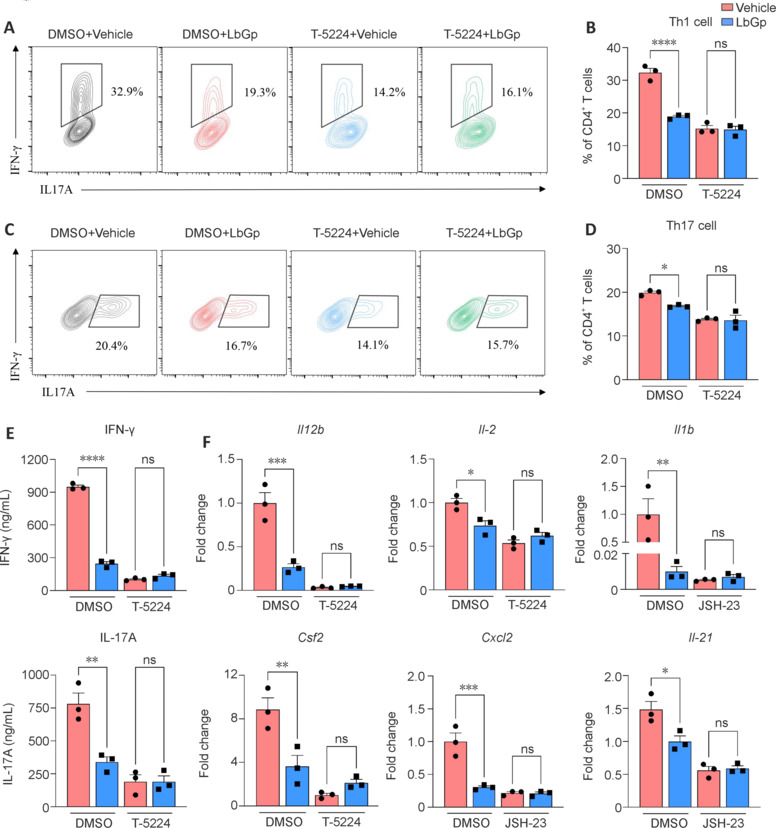
AP-1 inhibitor T-5224 blocks the inhibitory effect of LbGp on Th1/Th17 cell differentiation. Naïve CD4^+^CD62L^high^CD44^low^ T cells, purified via MACS, were pre-treated with or without T-5224 and subsequently cultured in Th1/Th17 polarization medium for 4–5 days with or without the addition of LbGp. (A–D) Flow cytometric analysis was conducted to assess the impact of LbGp on Th1/Th17 cell differentiation in the presence or absence of T-5224 pretreatment (*n* = 3 per group). (E) The concentrations of IFN-γ and IL-17A in cell supernatants were detected using ELISA (*n* = 3 per group). (F) Quantitative analysis of the mRNA levels of Th1/Th17 signature cytokines was performed via quantitative polymerase chain reaction (*n* = 3 per group). Error bars indicate the mean ± standard error of the mean. *P < 0.05, **P < 0.01, ***P < 0.001, ****P < 0.0001. AP-1: Activator protein-1; CD: cluster of differentiation; DMSO: dimethyl sulfoxide; ELISA: enzyme-linked immunosorbent assay; IFN-γ: interferon-γ; IL: interleukin; LbGp: *Lycium barbarum* polysaccharide; MACS: magnetic-activated cell sorting; ns: not significant; Th: T helper cell; Treg: regulatory T cell.

LbGp treatment significantly modulated key signaling pathways involved in the pathogenesis of EAE, including the NLRP3 inflammasome, NF-κB signaling pathway, and AP-1 signaling pathway (**Figure 5E** and **F**). Among these pathways, AP-1 has been confirmed as a key transcriptional regulator that integrates signals to drive gene expression for Th1/Th17 lineage commitment (Yamazaki et al., 2017; Schnoegl et al., 2023); it is thus a prime target for therapeutic intervention. Building on these insights, we sought to further explore the role of AP-1 in mediating the immunosuppressive effects of LbGp, specifically focusing on whether pretreatment with AP-1 inhibitor can block the suppressive effects of LbGp on Th1/Th17 cell differentiation. To test this hypothesis, we investigated the effects of the AP-1 inhibitor T-5224 on the capacity of LbGp to suppress Th1/Th17 differentiation *in vitro* (Yoshida et al., 2015). We pre-treated the cells with T-5224 before administering LbGp, and then polarized naïve T cells to Th1/Th17 lineages. T-5224 pretreatment effectively dismissed the suppressive effects of LbGp on Th1 and Th17 differentiation (**Figure 6A–D**). This blockade effect was further evidenced by the unchanged expression levels of IFN-γ and IL-17A in the cell supernatant (**Figure 6E**). Moreover, the inhibitory effects of LbGp on Th1/Th17-associated cytokine mRNA expression were completely abrogated when T-5224 was applied prior to LbGp treatment (**Figure 6F**). These data suggest that the therapeutic efficacy of LbGp may be critically dependent on its ability to inhibit the AP-1 signaling pathway.

## Discussion

The present study provides compelling evidence that LbGp possesses substantial therapeutic potential for treating EAE. LbGp administration significantly decreased Th1 and Th17 cell differentiation while promoting Treg polarization both *in vivo* and *in vitro*. The therapeutic effects of LbGp were linked to the inhibition of gene expression profiles and key signaling pathways involved in T cell activation and differentiation, especially the AP-1 pathway. Furthermore, pretreatment with the AP-1 inhibitor T-5224 effectively blocked the suppressive effects of LbGp on Th1 and Th17 cell differentiation. This finding indicates that LbGp may exert protective effects in EAE by modulating inflammatory responses and reducing pathogenic CD4^+^ T cell generation at least partially via the AP-1 signaling pathway. In summary, our results underscore the potential of LbGp as a novel therapeutic agent for MS and emphasize its capacity to modulate the pathogenic CD4^+^ T cell responses that contribute to the autoimmune and neuroinflammatory processes underlying the disease.

Given the limitations and safety concerns of current immunosuppressive therapies, specifically targeting pathogenic CD4^+^ T cells in MS remains an important challenge. Although agents such as natalizumab, fingolimod, and alemtuzumab have shown efficacy for reducing relapse rates, they are accompanied by important risks, including heightened susceptibility to opportunistic infections and severe adverse effects such as progressive multifocal leukoencephalopathy (Hauser and Cree, 2020; Luna et al., 2020). These challenges underscore the urgent need for more precise and safer therapeutic options. Together with existing literature, our study underscores the favorable tolerability and safety profile of LbGp. Unlike broad-spectrum immunosuppressants, LbGp selectively modulates pathogenic T cell responses without inducing extensive immunosuppression, thereby minimizing the risk of severe side effects. We observed that a therapeutic dose of LbGp over a 1-month period had no significant effects on body weight, leukocyte counts in central or peripheral immune organs, or the distribution of immune cell subsets in the peripheral blood of C57BL/6J mice. Similarly, Zhou et al. (2022) reported that C57BL/6J mice can tolerate oral doses of LbGp up to 100 mg/kg without adverse effects, and Wang et al. (2021) noted an absence of toxicity while investigating the immune-modulatory effects of LbGp in non-obese diabetic mice. Additionally, Li et al. (2024) observed that adolescents with subthreshold depression can tolerate oral doses as high as 300 mg/day, thereby reinforcing the favorable safety and tolerability profile of LbGp. Thus, LbGp appears to be a promising candidate for MS therapy, with an acceptable safety profile and great tolerability; however, further studies are necessary to assess its effects in different contexts and higher dosages.

In addition to its direct effects on pathogenic CD4^+^ T cells, LbGp may also offer therapeutic benefits by modulating peripheral myeloid cells, which are central to the maintenance and amplification of Th1/Th17 immune responses in the pre-onset stage of EAE (Lu et al., 2020; Zheng et al., 2020; Cao et al., 2022b). This process is mainly facilitated by cytokine secretion and co-stimulatory molecule expression, and is crucial for priming CD4^+^ T cells into pathogenic Th1/Th17 subsets (Li et al., 2019). By influencing peripheral myeloid cell functions, LbGp may mitigate the early inflammatory cascade, potentially halting disease progression before substantial neurological damage occurs. Recent studies support this hypothesis, highlighting the ability of LbGp to modulate myeloid cells. Wang et al. (2023) demonstrated that LbGp mitigates enteritis by modulating macrophage polarization, which in turn reduces the inflammatory response in a dextran sulfate sodium-induced colitis model. Furthermore, Ding et al. (2021) demonstrated that LbGp mitigates inflammation by inhibiting glycolysis and M1 macrophage differentiation, primarily through promoting the ubiquitination and subsequent downregulation of pyruvate kinase 2 (a key enzyme that regulates glycolytic metabolism in inflammatory macrophages). In our study, delayed EAE onset and the suppression of pathogenic CD4^+^ T cell priming after MOG restimulation suggest a potentially crucial role of peripheral myeloid cells in the therapeutic effects of LbGp. Notably, the discrepancy between the inhibitory effects of LbGp on induced Treg differentiation *in vitro* (data not shown) and its promotion of Treg polarization in MOG peptide restimulation assays and EAE models indicates that the therapeutic effects of LbGp may be mediated through its impact on the myeloid compartment rather than on direct interactions with T cells. In addition to its potential effects on peripheral myeloid cells, recent studies have demonstrated that LbGp inhibits inflammatory cytokine production in microglia and astrocytes, thereby reducing neuroinflammation and subsequent neurodegeneration in various neurological disease models (Jiang et al., 2023; Xu et al., 2024a). Future studies should directly investigate other immune cell types in relation to the therapeutic efficacy of LbGp; this might uncover additional mechanisms by which LbGp exerts anti-inflammatory effects in EAE.

The present study focused on the influence of LbGp on the NLRP3 inflammasome, NF-κB, and AP-1 signaling pathways in pathogenic CD4^+^ T cells through its immunomodulatory effects. These pathways are crucial for Th1/Th17/Treg function, which is central to MS pathogenesis. NLRP3 significantly influences CD4^+^ T cell subset differentiation and function, shaping their unique roles in immune responses. In Tregs, NLRP3 functions as a negative regulator by impeding FOXP3 expression, consequently reducing Treg generation and suppressive function (Park et al., 2019). In Th17 cells, NLRP3 is crucial for IL-1β production through a T cell-intrinsic inflammasome pathway, thus promoting Th17 cell survival and expansion in EAE (Martin et al., 2016). NLRP3 also facilitates Th1 differentiation by promoting IL-1β secretion through caspase-1 activation, which in turn drives IFN-γ production (Arbore et al., 2016). NF-κB signaling enhances the transcription of inflammatory mediators such as IL-1β, IL-12b, and IL-23, thereby promoting pathogenic CD4^+^ T cell differentiation and activation (Lalle et al., 2024).

AP-1 is a crucial transcription factor that is associated with the inflammatory activation of T cells and might interact with both the NLRP3 inflammasome and NF-κB pathways (Zhu et al., 2020; Hokello et al., 2021; Xu et al., 2024b). AP-1 forms through the dimerization of c-Fos and c-Jun and is activated by upstream signals such as mitogen-activated protein kinases (e.g., c-Jun N-terminal kinase and extracellular signal-regulated kinase) and cytokine receptors. Upon activation, AP-1 binds to the promoters of IL-2, TNF-α, and IFN-γ, which are crucial for the expansion and effector functions of Th1 and Th17 cells (Yukawa et al., 2020; Cobos Jiménez et al., 2023). AP-1 can also upregulate the expression of co-stimulatory molecules such as CD40L, further enhancing T cell activation and sustaining pathogenic responses (Renoux et al., 2020). In the present study, LbGp significantly inhibited AP-1 activity; this was a crucial mechanism for its therapeutic effects, as evidenced by rescue experiments using an AP-1 inhibitor. LbGp inhibits AP-1, thus disrupting the transcription of downstream pro-inflammatory genes and attenuating the activation and expansion of pathogenic Th1 and Th17 cells. This is particularly important because AP-1 serves as a pivotal node in the amplification of inflammatory signals, functioning as a downstream effector of NLRP3 activation and an intersection point with the NF-κB pathway. By targeting AP-1, LbGp effectively attenuates Th1/Th17 differentiation and promotes Treg polarization, thereby shifting the immune balance toward a more regulatory phenotype. This mechanistic insight enhances our understanding of the action of LbGp and underscores the importance of AP-1 as a therapeutic target in MS. It also highlights the potential of LbGp for modulating critical immune pathways, offering a novel approach for MS treatment through the selective targeting of molecular drivers involved in pathogenic CD4^+^ T cell responses.

Our results represent substantial advancements in the treatment of MS by providing the first evidence of the therapeutic efficacy of LbGp in EAE models. These results suggest that LbGp is a safe and well-tolerated therapeutic option that may effectively modulate the critical pathogenic immune responses implicated in MS. Additionally, we conducted RNA-seq of CD4^+^ T cells isolated from mice to study how LbGp treatment impacts the expression profiles of genes associated with T cell activation, inflammation, differentiation, and migration. The use of an AP-1 inhibitor in rescue experiments clarified the mechanisms underlying the therapeutic effects of LbGp, thereby suggesting targeted therapies with improved efficacy and safety for treating pathogenic CD4^+^ T cell-mediated diseases.

However, our study had certain limitations. First, there was a lack of dose-response experiments, which are essential for determining the optimal therapeutic range of LbGp. Without these data, it is difficult to fully characterize the therapeutic potential of LbGp and optimize its clinical application for different patient populations. Future studies should therefore prioritize a systematic exploration of various dosing regimens to identify the most effective and safest dosage for both short- and long-term use. Second, our study only evaluated LbGp in a single MS model. Although EAE is a widely accepted and robust model for studying MS, it does not encompass the full spectrum of disease complexity in humans. By testing the efficacy of LbGp in multiple MS models that capture different aspects of the disease, a more comprehensive understanding will be gained of how LbGp functions under diverse pathological conditions. Third, our research concentrated predominantly on immune cells and neuroinflammation, and we did not explore the effects of LbGp on oligodendrocytes or the remyelination process. This omission leaves an important aspect of MS pathophysiology unaddressed; oligodendrocyte loss and impaired remyelination are central to the neurodegenerative processes in MS. Future research should prioritize this knowledge gap by conducting in-depth studies to evaluate the effects of LbGp on oligodendrocyte survival and differentiation and myelin regeneration in the CNS (Lubetzki et al., 2020; Tepavčević and Lubetzki, 2022). Fourth, the LbGp used in our study was a composite formulation comprising five distinct glycopeptide components, designated LbGp1–5 (Wang et al., 2024). Although the combined action of these components has notable therapeutic potential for modulating immune responses, the specific role of each component remains unclear. Future research should therefore focus on the individual contribution of each component to the overall therapeutic effects of LbGp. Understanding the pharmacodynamics of each individual glycopeptide may also help to minimize unwanted side effects and optimize dosage regimens, ultimately improving patient outcomes and enhancing the safety profile of LbGp-based therapies.

In conclusion, our research comprehensively evaluated the therapeutic potential of LbGp in EAE. Our findings highlight LbGp as a promising agent for MS that may offer a safe and effective alternative to current immunosuppressive therapies. Nevertheless, further research is needed to translate these findings into clinical applications; this should focus on optimizing its effective dosage, exploring its long-term effects, and developing emerging drug delivery systems. Clinical trials will also be essential for evaluating the safety, efficacy, pharmacokinetics, immunotoxicity, and therapeutic impacts of LbGp in patients. An exploration of the use of LbGp in combination with other immunomodulatory drugs might also provide insights into its potential role in combination therapies. Ultimately, it is hoped that these efforts will enable LbGp to become a viable therapeutic option for MS while minimizing the risks associated with current treatments.

## Additional files:

***Additional Figure 1:*** *Effect of different LbGp dosages on the severity of EAE.*

Additional Figure 1 Effects of different LbGp dosages on the severity of EAE.
The effects of different LbGp dosages on the disease progression curve of EAE mice (*n* = 8 per group). Error bars indicate the mean ± standard error of the mean. Statistical analyses were performed using two-way analysis of variance followed by the Bonferroni test. ****P < 0.0001. EAE: Experimental autoimmune encephalomyelitis; LbGp: *Lycium barbarum* polysaccharide; ns: no significance.

***Additional Figure 2:*** *Evaluation of the long-term safety and tolerability of LbGp.*

Additional Figure 2 Evaluation of the long-term safety and tolerability of LbGp.
Mice were administered 5 mg/kg of LbGp or an equivalent volume of saline via gavage once daily for 4 weeks. (A) Body weight change curves over the 4-week period for the different groups (*n* = 5 per group). (B) Absolute cell counts of CD45+ cells in the blood, inguinal lymph nodes, spleen, and thymus of mice receiving the indicated treatments (*n* = 3 per group). (C–F) Representative flow cytometric plots and statistical results showing the percentages of immune cell subtypes in the blood (*n* = 5 per group). (G) H&E staining of the liver, kidney, spleen, and inguinal lymph nodes from mice treated with normal saline or LbGp for 4 weeks. There were no histopathological differences in the liver, kidney, spleen, or inguinal lymph nodes between LbGp- and normal saline-treated mice. Error bars indicate the mean ± standard error of the mean. Statistical analyses were performed using unpaired Student’s *t*-tests. CD: Cluster of differentiation; H&E: hematoxylin and eosin; LbGp: *Lycium barbarum* polysaccharide; ns: not significant.

***Additional Figure 3:*** *Effects of LbGp on the viability of CD4*^*+*^ *T cells.*

Additional Figure 3 Effects of LbGp on the viability of CD4^+^ T cells.
MACS-purified CD4^+^ T cells were cultured in 96-well plates with the specified compounds for 3 days (*n* = 3 per group). (A) AV and PI staining were used to differentiate between live (PI^-^AV^-^), apoptotic (PI^-^AV^+^), and necrotic/dead (PI^+^AV^+^) cells under vehicle or LbGp treatment conditions. Error bars indicate the mean ± standard error of the mean. Statistical analyses were performed using unpaired Student’s *t*-tests. AV: Annexin V; CD: cluster of differentiation; LbGp: *Lycium barbarum* polysaccharide; MACS: magnetic-activated cell sorting; ns: no significance; PI: propidium iodide.

## Data Availability

*All relevant data are within the paper and its Additional files*.
