# Supplementary figures and images for: Effects of Lycium barbarum polysaccharide on the activation of pathogenic CD4+ T cells in a mouse model of multiple sclerosis
Source: Neural Regen Res. 2025 Mar 25;21(6):2563–72. doi: 10.4103/NRR.NRR-D-24-01093 (PMC13211841; doi:10.4103/NRR.NRR-D-24-01093)

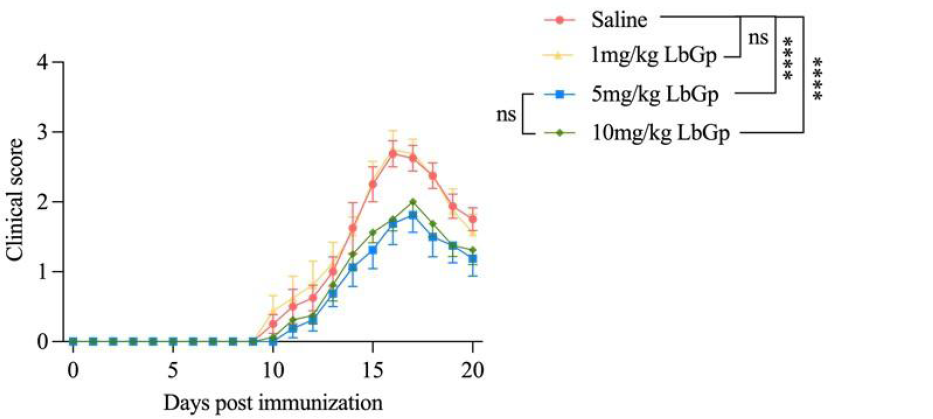

Supplement: Supplementary file 1 [file NRR-21-2563_Suppl1.tif]

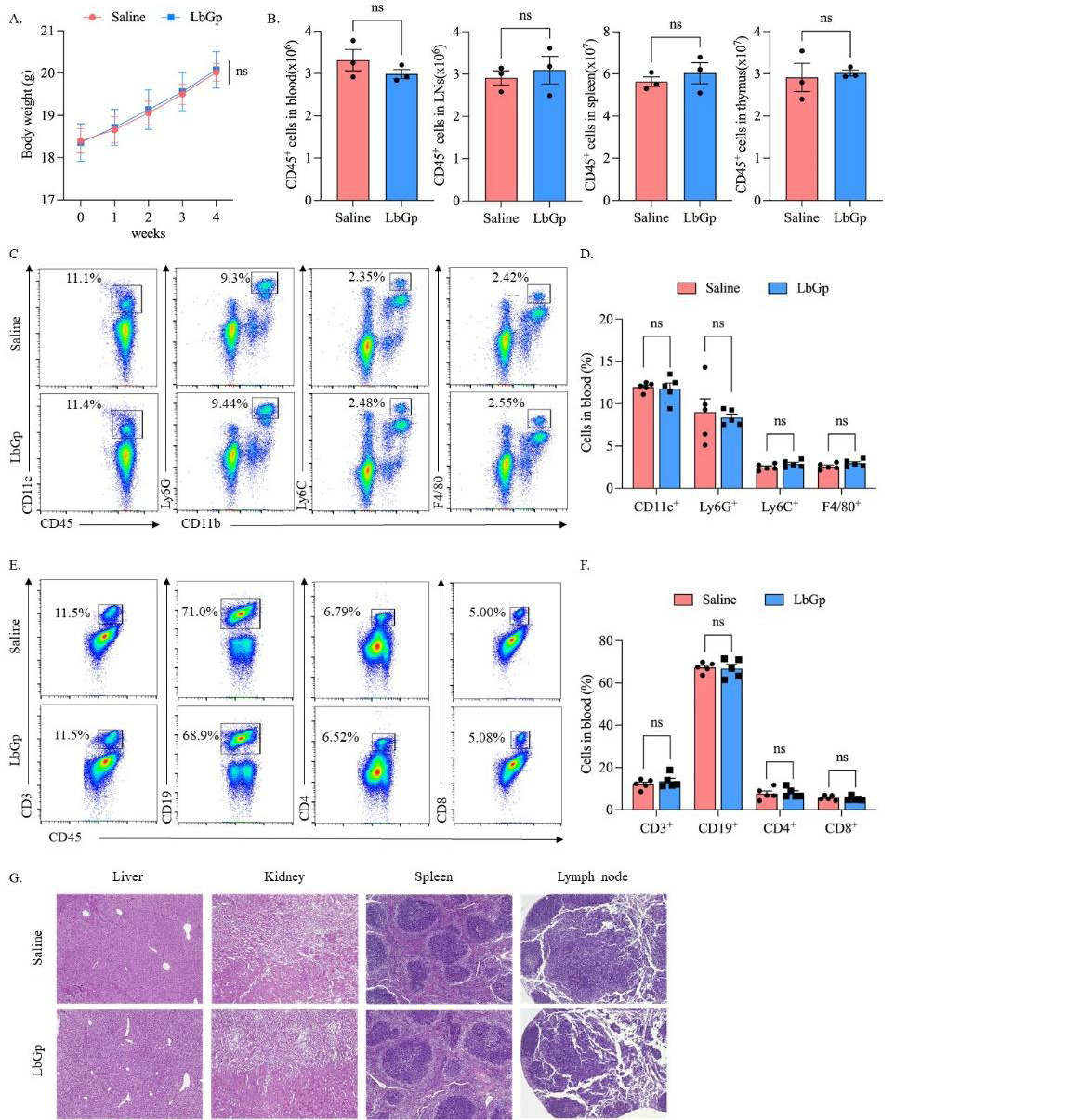

Supplement: Supplementary file 2 [file NRR-21-2563_Suppl2.tif]

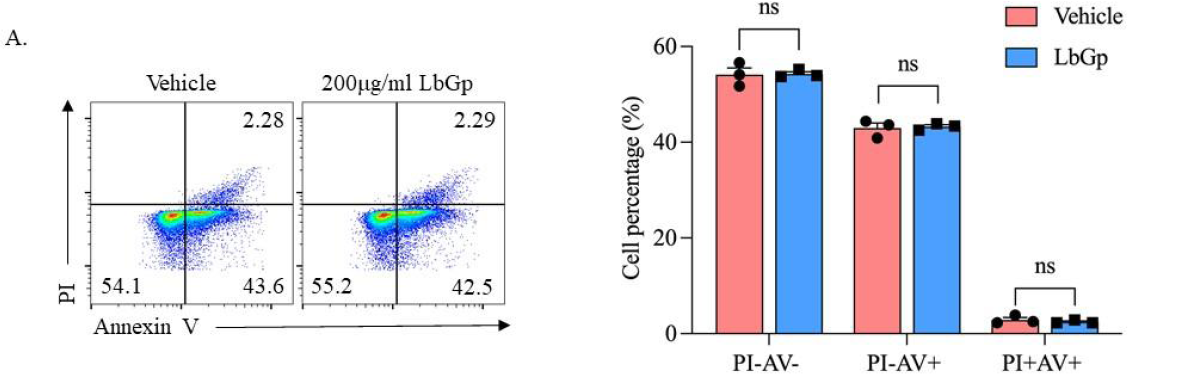

Supplement: Supplementary file 3 [file NRR-21-2563_Suppl3.tif]
